# Supplementary material for: Glucose Decoration on Wall Teichoic Acid Is Required for Phage Adsorption and InlB-Mediated Virulence in Listeria ivanovii
Source: J Bacteriol. 2021 Jul 22;203(16):e00136-21. doi: 10.1128/JB.00136-21 (PMC8297528; doi:10.1128/JB.00136-21)
Supplement: Supplemental file 1 — Fig. S1 and Tables S1 and S2. Download JB00136-21_Supp_1_seq4.pdf, PDF file, 0.2 MB [file jb00136-21_supp_1_seq4.pdf]

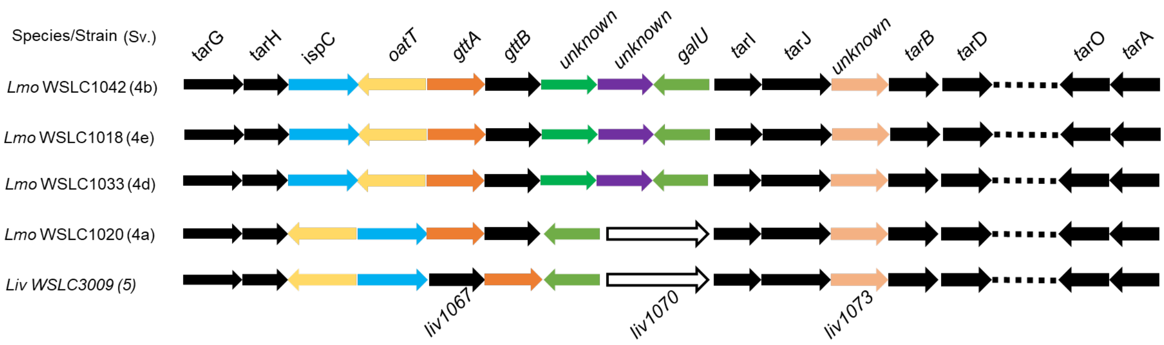


**Figure S1**. Comparison of genes encoding type II WTA biosynthesis pathways in the indicated *Listeria* serovars and strains.

Table S1. Homologs of Liv1070 proteins identified in strains from various of *Listeria* subspecies (only top 20 are shown).

| Description | Scientific Name | Query Cover | E value | Per. ident | Acc. Len | Accession |
|---|---|---|---|---|---|---|
| bifunctional glycosyltransferase family 2 protein/CDP-glycerol:glycerophosphate glycerophosphotransferase | Listeria ivanovii | 100% | 0.00E+00 | 100 | 783 | WP_014092498.1 |
| bifunctional glycosyltransferase family 2 protein/CDP-glycerol:glycerophosphate glycerophosphotransferase | Listeria ivanovii | 100% | 0.00E+00 | 99.87 | 783 | WP_111124699.1 |
| MULTISPECIES: bifunctional glycosyltransferase family 2 protein/CDP-glycerol:glycerophosphate glycerophosphotransferase | Listeria | 100% | 0.00E+00 | 97.06 | 783 | WP_003719308.1 |
| CDP-glycerol glycerophosphotransferase family protein | Listeria ivanovii | 100% | 0.00E+00 | 96.81 | 783 | WP_185634543.1 |
| CDP-glycerol glycerophosphotransferase family protein | Listeria seeligeri | 100% | 0.00E+00 | 96.55 | 783 | WP_194329958.1 |
| bifunctional glycosyltransferase family 2 protein/CDP-glycerol:glycerophosphate glycerophosphotransferase | Listeria seeligeri | 100% | 0.00E+00 | 96.3 | 783 | WP_139590672.1 |
| CDP-glycerol glycerophosphotransferase family protein | Listeria seeligeri | 100% | 0.00E+00 | 94.13 | 783 | WP_181249218.1 |
| CDP-glycerol glycerophosphotransferase family protein | Listeria seeligeri | 100% | 0.00E+00 | 94 | 783 | WP_194341205.1 |
| CDP-glycerol glycerophosphotransferase family protein | Listeria seeligeri | 100% | 0.00E+00 | 93.87 | 783 | WP_185365227.1 |
| CDP-glycerol glycerophosphotransferase family protein | Listeria seeligeri | 100% | 0.00E+00 | 93.87 | 783 | WP_185338197.1 |
| CDP-glycerol glycerophosphotransferase family protein | Listeria welshimeri | 100% | 0.00E+00 | 91.95 | 783 | WP_185303904.1 |
| unnamed protein product | | 100% | 0.00E+00 | 91.95 | 783 | WP_194342659.1 |
| CDP-glycerol glycerophosphotransferase family protein | Listeria welshimeri | 100% | 0.00E+00 | 91.95 | 783 | WP_194355605.1 |
| CDP-glycerol glycerophosphotransferase family protein | Listeria welshimeri | 100% | 0.00E+00 | 91.95 | 783 | WP_194352431.1 |
| glycosyltransferase | Listeria welshimeri | 100% | 0.00E+00 | 91.83 | 783 | MBC2346148.1 |
| glycosyltransferase | Listeria welshimeri | 100% | 0.00E+00 | 91.83 | 783 | MBC1249545.1 |
| glycosyltransferase | Listeria welshimeri | 100% | 0.00E+00 | 91.83 | 783 | MBC1611992.1 |
| glycosyltransferase | Listeria welshimeri | 100% | 0.00E+00 | 91.83 | 783 | MBC1451016.1 |
| CDP-glycerol glycerophosphotransferase family protein | Listeria welshimeri | 100% | 0.00E+00 | 91.83 | 783 | WP_194338958.1 |

Table S2. Strains, plasmids, and primers.

| **Strain** | Species | Source |
|---|---|---|
| WSLC 3009 | *L. ivanovii subsp. ivanovii* | Ref. (1) |
| 3009Δ*liv1070* | *L. ivanovii subsp. ivanovii* | This study |
| 3009Δ*liv1070*::pPL2(*liv1070*) | *L. ivanovii subsp. ivanovii* | This study |
| 1042 | *L. monocytogenes* | Ref. (2) |
| 1042Δ*gttA* | *L. monocytogenes* | Ref. (2) |
| XL-1 blue | *E. coli* | Thermofisher |
| **Plasmid** | Description | Source |
| pHoss1 | Ampicillin (gram-negative), erythromycin (gram-positive) resistance gene | Ref. (3) |
| **Primer** | Sequence | Purpose |
| pHoss1_fwd2 | TTACGAACCTCTTTTGTTC | Synthesis of pHoss backbone |
| pHoss1_rev2 | CAAGATTTTCTTCACACTAGC | Synthesis of pHoss backbone (complement) |
| 1070 (3009) up_fwd | ACTAACATTGCTAGTGTGAAGAAAATCTTGTTGCTTGAAGAATCGCATC | Synthesis of up upstream flanking region C |
| 1070 (3009) up_rev | TTTAAAAGCTAAATCGCTTAATCTCCCTTTGTAC | Synthesis of up upstream flanking region C (complement) |
| 1070 (3009) down_fwd | AAAGGGAGATTAAGCGATTTAGCTTTTAAAATGTGGTATAATAAAC | Synthesis of up downstream flanking region D |
| 1070 (3009) down_rev | GAACTTCTTTAGAACAAAAGAGGTTCGTAATATGGTTACTTGATTCAACAATC | Synthesis of up upstream flanking region D (complement) |


1. Hupfeld M, Trasanidou D, Ramazzini L, Klumpp J, Loessner MJ, Kilcher S. 2018. A functional type II-A CRISPR-Cas system from Listeria enables efficient genome editing of large non-integrating bacteriophage. Nucleic Acids Res 46:6920-6933.
2. Sumrall ET, Shen Y, Keller AP, Rismondo J, Pavlou M, Eugster MR, Boulos S, Disson O, Thouvenot P, Kilcher S, Wollscheid B, Cabanes D, Lecuit M, Grundling A, Loessner MJ. 2019. Phage resistance at the cost of virulence: Listeria monocytogenes serovar 4b requires galactosylated teichoic acids for InlB-mediated invasion. PLoS Pathog 15:e1008032.
3. Abdelhamed H, Lawrence ML, Karsi A. 2015. A novel suicide plasmid for efficient gene mutation in Listeria monocytogenes. Plasmid 81:1-8.
